# High Sporozoite Antibody Titers in Conjunction with Microscopically Detectable Blood Infection Display Signatures of Protection from Clinical Malaria

**DOI:** 10.3389/fimmu.2017.00488

**Published:** 2017-05-08

**Authors:** Vittoria Offeddu, Ally Olotu, Faith Osier, Kevin Marsh, Kai Matuschewski, Vandana Thathy

**Affiliations:** ^1^Parasitology Unit, Max Planck Institute for Infection Biology, Berlin, Germany; ^2^Wellcome Trust Research Programme, Centre for Geographic Medicine Research-Coast, Kenya Medical Research Institute (KEMRI), Kilifi, Kenya; ^3^Centre for Tropical Medicine and Global Health, Nuffield Department of Medicine, University of Oxford, Oxford, UK; ^4^Molecular Parasitology, Institute of Biology, Humboldt University, Berlin, Germany

**Keywords:** malaria, *Plasmodium falciparum*, sporozoites, humoral immunity, clinical malaria, protective immunity

## Abstract

Immunoepidemiological studies typically reveal slow, age-dependent acquisition of immune responses against *Plasmodium falciparum* sporozoites. Naturally acquired immunity against preerythrocytic stages is considered inadequate to confer protection against clinical malaria. To explore previously unrecognized antisporozoite responses, we measured serum levels of naturally acquired antibodies to whole *Plasmodium falciparum* sporozoites (*Pf*spz) and the immunodominant (NANP)_5_ repeats of the major sporozoite surface protein, circumsporozoite protein, in a well-characterized Kenyan cohort. Sera were sampled at the start of the malaria transmission season, and all subjects were prospectively monitored for uncomplicated clinical malaria in the ensuing 6 months. We used Kaplan–Meier analysis and multivariable regression to investigate the association of antisporozoite immunity with incidence of clinical malaria. Although naturally acquired humoral responses against *Pf*spz and (NANP)_5_ were strongly correlated (*p* < 0.0001), 37% of *Pf*spz responders did not recognize (NANP)_5_. The prevalence and magnitude of antisporozoite responses increased with age, although some high *Pf*spz responders were identified among children. Survival analysis revealed a reduced risk of and increased time to first or only episode of clinical malaria among *Pf*spz or (NANP)_5_ responders carrying microscopically detectable *Plasmodium falciparum* (*Pf*) parasitemia at the start of the transmission season (*p* < 0.03). Our Cox regression interaction models indicated a potentially protective interaction between high anti-*Pf*spz (*p* = 0.002) or anti-(NANP)_5_ (*p* = 0.001) antibody levels and microscopically detectable *Pf* parasitemia on the risk of subsequent clinical malaria. Our findings indicate that robust antisporozoite immune responses can be naturally acquired already at an early age. A potentially protective role of high levels of anti-*Pf*spz antibodies against clinical episodes of uncomplicated malaria was detected, suggesting that antibody-mediated preerythrocytic immunity might indeed contribute to protection in nature.

## Introduction

Malaria transmission by *Plasmodium*-infected *Anopheles* mosquitoes results in at least 200 million malaria cases every year, and the majority of disease burden is caused by *Plasmodium falciparum* (*Pf*) infections in children (1).

During a blood meal, an infected mosquito typically injects fewer than 40 motile sporozoites into the human skin (2). After injection, a proportion of sporozoites reaches a blood capillary by active intradermal migration, breaches the endothelial cells of the blood vessel, and is passively transported to the liver through the blood circulation. Following a massive replication phase in hepatocytes, tens of thousands of liver merozoites are released to initiate asexual parasite propagation in erythrocytes, the only phase in the parasite life cycle causing malaria symptoms.

When experimentally inoculated in repeated high doses, arrested, but metabolically active, *Plasmodium* sporozoites are able to elicit protective and often lasting and sterile immune responses against homologous challenge in humans (3–5). Because the preerythrocytic phase of the life cycle is clinically silent, humans can be inoculated with high doses of sporozoites, providing a tantalizing rationale for preerythrocytic malaria vaccine approaches (6). *Plasmodium* sporozoites are considered immunologically silent in natural infections, because they are mostly inoculated in small numbers and are exposed to the immune system only for a brief period (7, 8).

Naturally acquired immune responses against whole *Pf* sporozoites (*Pf*spz) typically reveal age- and transmission-dependent increases in prevalence (9–11). Strong humoral responses are directed against the central repeat region (CRR) of the major sporozoite surface protein, circumsporozoite protein (CSP) (12–17). The CRR is composed of a varying number of NANP repeats, and a single *Pf* sporozoite inoculation is sufficient to trigger anti-NANP antibody responses (15, 18). However, in virtually all populations studied, a varying proportion of naturally exposed individuals remains unresponsive to NANP repeats, even after repeated exposures (19).

In addition to NANP repeats, immune responses can also target regions in the amino- or carboxy-termini of CSP  (20–22) or additional sporozoite antigens, such as thrombospondin-related anonymous protein (23–25) or sporozoite threonine–asparagine-rich protein (26, 27). However, no single preerythrocytic antigen has yet been identified that serves as a correlate of naturally acquired protection against infection or clinical disease. Rather, immunoepidemiological studies show that the breadth of preerythrocytic antigens recognized by an individual is associated with a lower risk of and an extended time to reinfection (28, 29).

In this study, we analyzed humoral immune responses against *Pf*spz in a cohort of naturally exposed individuals in Kenya. We compared prevalence and titers of antibodies against whole *Pf*spz and NANP repeats of CSP and assessed potentially protective contributions of antisporozoite immunity against clinical malaria. We show previously unrecognized high anti-*Pf*spz responses in children, which are not captured by (NANP)_5_-enzyme-linked immunosorbent assay (ELISA), and demonstrate potentially protective interactions between high levels of anti-*Pf*spz responses and asymptomatic *Pf* slide positivity at the time of serum collection on the risk of clinical malaria.

## Materials and Methods

### Study Design and Cohort

This was a follow-up study from seroepidemiological cohort studies previously conducted in Chonyi village (Kilifi County, Kenya) (30–34). At the time the study was conducted, this region experienced year-round moderate malaria transmission (~20–100 infective bites/person/year), with a short rainy season from November to December and an extended one from June to August causing two seasonal peaks in transmission (35). Details of the cohort and study area have been published previously (36). For this study, serum samples of 514 individuals aged 1.6 months–82 years were analyzed from the cross-sectional bleed conducted in October 2000, immediately prior to the start of the malaria transmission season. At the time of this cross-sectional bleed, no individual experienced clinical malaria episodes, but 37% carried a microscopically detectable asymptomatic *Pf* blood stage infection, as assessed by thick and thin blood films. Asymptomatic infection was defined as any microscopically detectable parasitemia in an afebrile individual. We measured serum levels of antibodies to (i) whole *Pf*spz and (ii) the (NANP)_5_ repeats of CSP. Active weekly surveillance and passive case detection for development of clinical malaria during a 26-week follow-up permitted a retrospective analysis of potential associations of humoral antisporozoite immune responses with incidence of clinical malaria, which was defined according to the following age-specific criteria: for infants <1-year old and for older children and adults (≥15 years old), an axillary temperature of ≥37.5°C plus any parasitemia; for children aged 1–14 years, an axillary temperature of ≥37.5°C, combined with a parasitemia of ≥2,500 parasites/μl (36).

### Generation of *Pf* Sporozoites

*Plasmodium falciparum* gametocyte cultures were derived from high parasitemic (5–8%) asexual *Pf*NF54 parasites, cultured in pooled human O+ red blood cells and RPMI-HEPES medium, supplemented with hypoxanthine, sodium carbonate, l-glutamine, and pooled, non-immune, heat-inactivated human A+ serum. The asexual *Pf* culture was diluted to 1% parasitemia into culture flasks containing 6 ml prewarmed medium, resulting in a final hematocrit of 5%, and gas flushed with 5% O_2_ and 5% CO_2_. Medium was replaced daily. From day 14 onward, cultures were examined daily for stage V gametocytemia, male:female ratio, and exflagellation rates. Infection of *Anopheles stephensi* mosquitoes was done by membrane feeding for 15 min at 26°C in the dark. Mosquitoes were maintained at 26°C and 80% humidity. Salivary glands were liberated between days 18 and 20 after infection by hand dissection in RPMI/3% BSA. Salivary glands were gently ground in a test tube, and the sporozoite suspension was centrifuged at 20 × *g* for 3 min to remove mosquito tissue debris. The mean sporozoite number per mosquito was 7,400. Two thousand to four thousand sporozoites per well were added to eight-well chamber slides (Medco), air dried, and stored at room temperature.

### Detection of Anti-*Pf*spz Antibodies by Immunofluorescence Assay (IFA)

For fixation, sporozoite slides were dipped in cold acetone for 1 min and rehydrated in PBS for 30 min. All incubation steps were done in a dark humid chamber for 1 h at 37°C. Blocking was done with PBS/3% BSA. After washing with PBS/0.5% BSA, human sera were added in serial dilutions ranging from 1:50 to 1:24,300 in PBS/0.5% BSA, followed by extensive washing with PBS/0.5% BSA. AlexaFluor488 goat anti-human IgG (1:500; Invitrogen) and Hoechst (1:1,000; Invitrogen) were used as secondary fluorescent antibody and nuclear stain, respectively. Slides were sealed after addition of Fluoromount (Southern Biotech) and analyzed by fluorescence microscopy. For the negative control, one well per slide was stained with pooled sera from 20 unexposed individuals from the United Kingdom. The pooled negative control sera did not deliver any IFA signal detectable by visual examination even at the lowest dilution. Cohort sera displaying a comparable response were considered to be IFA negative or unresponsive to *Pf*spz. For the positive control, one well per slide was stained with an anti*-Pf*CSP antibody (2A10) (37) or a Kenyan adult hyper immune serum. All IFA slides were randomized prior to microscopic screening to avoid biased evaluation, and arbitrary sample batches were repeated to exclude day-to-day variation of the assay. The reciprocal antisporozoite end titer was defined as the last dilution at which immunofluorescence was detectable by fluorescence microscopy.

### Detection of Serum Antibodies by ELISA

To quantify serum antibody titers against the CRR of CSP, a standard ELISA was performed using the (NANP)_5_-icosapeptide (Peptides and Elephants, Potsdam, Germany). A peptide stock solution (1 µg/µl) was prepared in sodium carbonate buffer (pH 9.3) and stored at −80°C. For experiments, the stock solution was diluted to a concentration of 400 ng/100 μl. F96 MaxiSorp plates (Nunc) were coated with peptide overnight at 4°C (400 ng/well). After extensive washing with PBS/0.05% Tween, unspecific binding was blocked with 100 µl casein (Thermo Scientific Blocker). After an additional wash, human sera (1:200 in 100 µl casein) were incubated for 1 h at 37°C. After extensive washing, plates were incubated for 1 h at 37°C with horseradish peroxidase-conjugated rabbit anti-human IgG (Dako Ltd.) at a 1:3,000 dilution in 100 µl casein. Following washing, bound human IgG was measured with OPD substrate (Sigmafast). The reactions were stopped with 50 µl 2 M H_2_SO_4_, and absorbance was read in an ELISA plate reader at 492 nm. To assess the variability of the ELISA assay, all sera were tested in duplicates, and the coefficient of variation was calculated for each paired serum sample. Samples that varied by ≥20% were tested again. A hyper immune Kenyan adult serum was used in duplicates on each ELISA plate as a positive control. The ratio of mean optical densities (ODs) of the positive serum samples was computed to assess plate to plate and day-to-day variability. For ratios ranging between 1.1 and 1.3 or 0.7 and 0.89, OD values were multiplied to adjust the data to a reference plate. For ratios <0.7 or >1.3, the experiment was repeated. Twenty sera from unexposed individuals from the United Kingdom were used as negative controls. The mean OD plus three SDs from all negative controls was determined as a cutoff for (NANP)_5_ seropositivity. ELISAs to measure serum levels of antibodies against *Pf* schizont extract and blood stage merozoite antigens were described previously (31).

### Statistical Analysis

A Spearman’s correlation was run to assess the relationship between ELISA ODs and IFA end titers. The Kruskal–Wallis test was applied for non-parametric analyses of variance in levels of antisporozoite antibodies across age groups. The Pearson’s χ^2^ test was used to determine the statistical significance of differences between proportions. The Mann–Whitney test determined statistically significant differences in antibody titer between two subsets of individuals. A binomial general linear model was used to calculate risk ratios in univariate analysis. Kaplan–Meier survival analysis and the log-rank Mantel–Cox test were used to compare the incidence of clinical malaria in distinct subsets of the cohort.

For multivariable analyses, we used a fractional polynomial Cox regression model that best predicts the time to first or only episode of clinical malaria for individuals with different levels of antisporozoite antibodies and predicted the log relative hazard against increasing levels of antibodies. Stepwise regression was conducted to select the most parsimonious set of covariates that are best predictors of clinical malaria. The variables with least significant coefficients (*p* > 0.2) were dropped one at a time until the final model was obtained. Likelihood ratio tests were run to compare the fit of the models with and without each variable. The final model was adjusted for the following confounders: age, plasma levels of antibodies against blood stage antigens [merozoite surface proteins 1-3 (MSP1-3), apical membrane antigen-1 (AMA1), erythrocyte binding antigen 175 (EBA-175), and *Pf* schizont extract], and malaria exposure, or unweighted local malaria prevalence index, as measured by the proportion of infected individuals within a 1 km radius of the index case. The malaria exposure index was calculated over the 6-month follow-up period using weekly slide data for acute episodes only (active case detection) and using the cross-sectional slide data (38).

To examine the effect of interactions between baseline antisporozoite antibody levels and microscopically detectable *Pf* parasitemia on malaria risk, hazard ratios were computed through a Cox regression model with and without one-, two-, or three-way interactions between antisporozoite antibody levels, microscopically detectable *Pf* infection, and malaria exposure index. Models with and without interactions were compared using the log likelihood ratio test.

The fractional polynomial Cox regression model and the interaction model are presented using anti-(NANP)_5_ ODs as a continuous variable. To reduce the skewness while maintaining the direction of association, anti-(NANP)_5_ OD values were transformed to negative inverse values before running the models.

Data were compared for statistically significant differences at a significance level of α = 0.05. The Bonferroni correction was applied to control for the increasing familywise error rate through multiple comparisons. Four families of statistical tests were defined based on the common dependent variable and null hypothesis tested. For each family composed of *k* significance tests, a new critical value was defined as α/*k*. Individual tests within these families were considered significant at α = 0.05 level if *p* < (α/*k*). The following families were defined:

1. Association of anti-*Pf*spz or anti-(NANP)_5_ antibody prevalence or titers with age; *k* = 8; α_new_ = 0.05/8 = 0.006.
2. Association of anti-*Pf*spz or anti-(NANP)_5_ antibody prevalence or titers with blood film status (for each separate age category), *k* = (4 tests × 9 age categories) = 36; α_new_ = 0.05/36 = 0.001.
3. Association of anti-*Pf*spz or anti-(NANP)_5_ antibody levels with incidence of clinical malaria; *k* = 4; α_new_ = 0.05/4 = 0.013.
4. Interaction of anti-*Pf*spz or anti-(NANP)_5_ antibody levels with blood film positivity status and association with incidence of clinical malaria; *k* = 14; α_new_ = 0.05/14 = 0.004.

All statistical analyses were performed using STATA/SE12 (StataCorp).

## Results

### Incidence of Clinical Malaria

Overall, the risk of developing at least one episode of clinical malaria during 6 months of follow-up decreased with age (*p* < 0.0001), with an overall rate of 0.4 episodes/person/year (Table S1 in Supplementary Material).

Asymptomatic *Pf* blood stage infection at the beginning of the malaria transmission season was detected in 37% of individuals, although this proportion was higher in younger age groups (3–15 years) (Table S1 in Supplementary Material). One or more malaria episodes were reported in 22% of blood film-positive individuals (Table S1 in Supplementary Material). This proportion was significantly lower (11%) among subjects who were blood film negative at the beginning of the observation period (*p* = 0.001) (Table S1 in Supplementary Material). This group may include parasite-free individuals and subjects carrying *Pf* parasites below the detection threshold by microscopy.

### Naturally Acquired Antibody Responses against *Pf* Sporozoites

**Figure 1:**
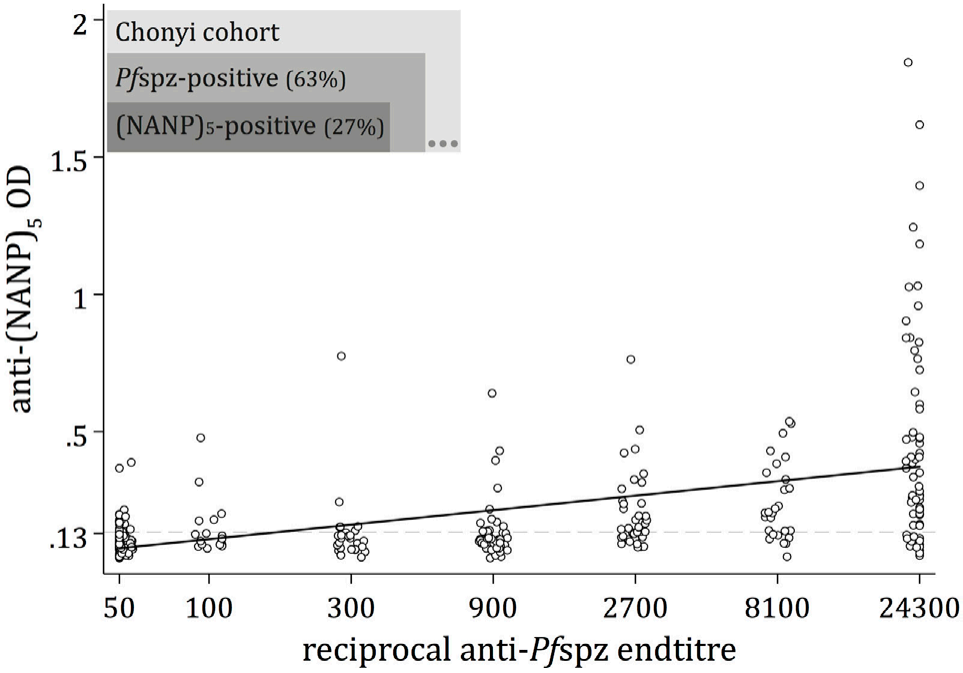
**Naturally acquired antibody responses against *Plasmodium falciparum (Pf)* sporozoites**. Antibody responses to whole *Pf* sporozoites (*Pf*spz) and to the central repeat region of the circumsporozoite protein were measured by immunofluorescence assay (IFA)  on air-dried *Pf*spz and enzyme-linked immunosorbent assay (ELISA) using (NANP)_5_ peptide, respectively. Anti-*Pf*spz end titers were defined as the last dilution at which sera recognized *Pf*spz by IFA. Anti-*Pf*spz antibody titers are log transformed. The (NANP)_5_-ELISA seropositivity cutoff (dashed line) was defined as the mean optical density (OD) plus 3 SDs of 20 sera from unexposed individuals (OD = 0.135). Serum from a hyperimmune Kenyan adult (20H) served as a positive control; black line: fitted regression line; Spearman’s ρ = 0.668; *p* < 0.0001.

A substantial fraction (63%) of the Chonyi sera recognized air-dried, whole *Pf*spz by IFA (Figure 1). The proportion of anti-(NANP)_5_ responders was substantially lower (27%), and all (NANP)_5_-reactive individuals except three were also classified as *Pf*spz responders (Figure 1). A large subset (36%) of sera recognized *Pf*spz but not (NANP)_5_. We observed an overall correlation of increased intensity of (NANP)_5_ responses in individuals with high anti-*Pf*spz titers (*p* < 0.0001) (Figure 1). However, even among the sera that produced the highest *Pf*spz reactivities by IFA, some remained (NANP)_5_ negative (Figure 1).

**Figure 2:**
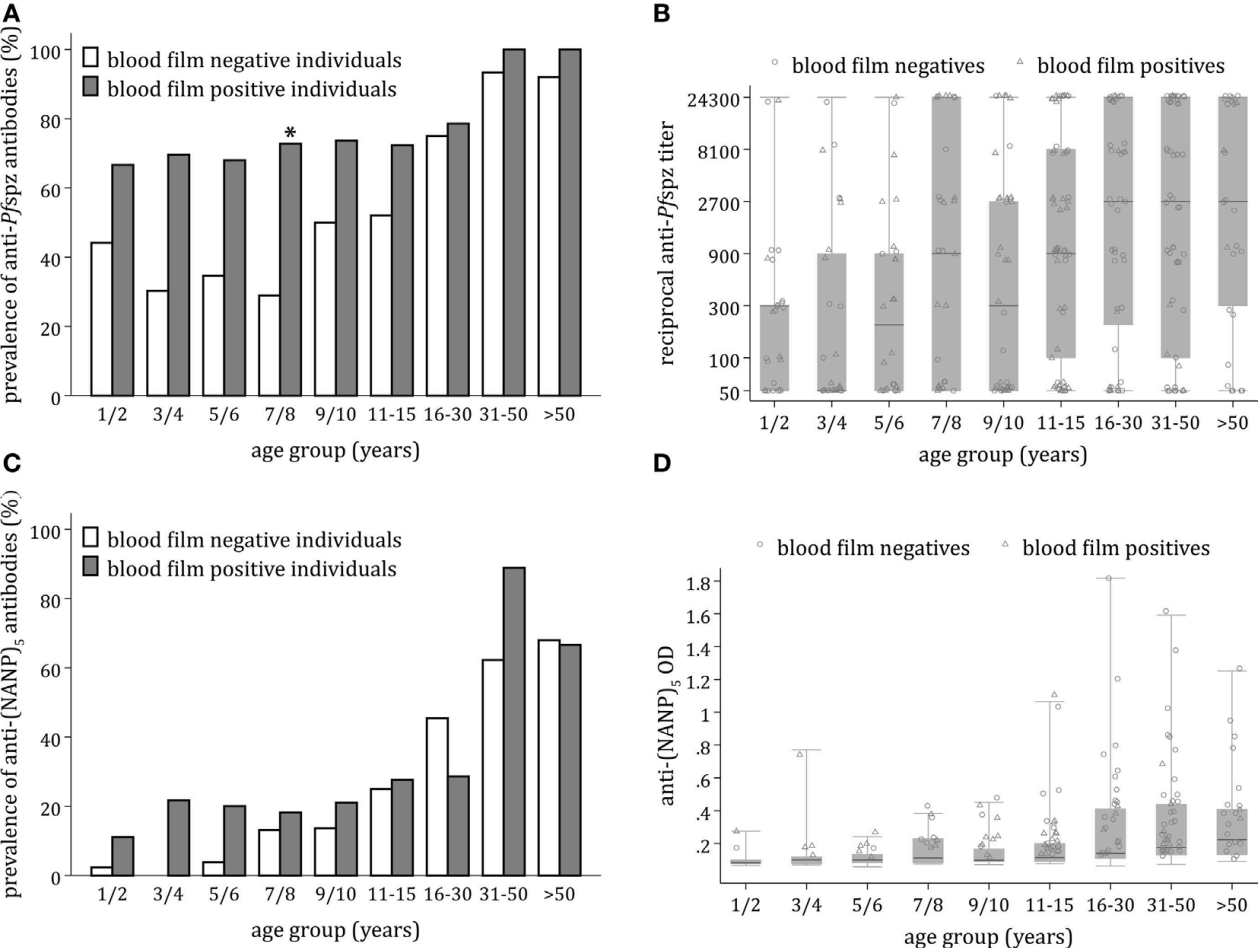
**Age-dependent acquisition of antisporozoite antibodies and correlation with microscopically detectable *Plasmodium falciparum (Pf)*  infection**. **(A)** Proportion of sera responding to *Pf* spzorozoites (*Pf*spz) by immunofluorescence assay (IFA) in each age group, stratified by blood film positivity. Anti-*Pf*spz antibody prevalence was significantly correlated with age among blood film-negative individuals (Pearson’s χ^2^ = 73.2; *p* < 0.001), but not blood film-positive individuals (Pearson’s χ^2^ = 5.4; *p* = 0.719). In all age groups, the proportion of responders was higher among blood film-positive individuals, but this was only significant among young individuals aged 7–8 years (Pearson’s χ^2^ = 10.8; *p* = 0.001). **(B)** Distribution of anti-*Pf*spz antibody titers by age category, excluding IFA-non-responders. Anti-*Pf*spz antibody titers are plotted on a log scale. Median anti-*Pf*spz antibody titers increased significantly with increasing age (Kruskal–Wallis test: χ^2^ = 32.7; *p* < 0.001). Each serum was tested in threefold serial dilutions (1:50–1:24,300). The number of high responders was detected in all age groups. Within individual age groups, differences in antibody levels between blood film-positive individuals and blood film-negative individuals were non-significant (Mann–Whitney test: *p* > 0.05). Boxes and horizontal bars indicate the 75th and 25th percentiles and median, respectively. Circles and triangles represent individual sera of blood film-negative or blood film-positive individuals, respectively. Whiskers represent minimum and maximum values. **(C)** Proportion of sera responding to (NANP)_5_ by enzyme-linked immunosorbent assay (ELISA) in each age group, stratified by blood film positivity. The proportion of (NANP)_5_-seropositive sera increased with age in both blood film-negative subjects (Pearson’s χ^2^ = 95.9; *p* < 0.001) and blood film-positive individuals (Pearson’s χ^2^ = 23.9; *p* = 0.002). Within each age group, the proportion of responders was similar in blood film-positive individuals and blood film-negative individuals. **(D)** Distribution of anti-(NANP)_5_ antibody titers by age category. There was an age-dependent increase in median anti-(NANP)_5_ titers, but this was not statistically significant (Kruskal–Wallis test: χ^2^ = 11.2; *p* = 0.193). Differences in anti-(NANP)_5_ antibody levels among blood film-positive individuals or blood film-negative individuals were non-significant in all age groups (Mann–Whitney test: *p* < 0.05); dashed line: ELISA seropositivity cutoff = 0.135. Boxes and horizontal bars indicate the 75th and 25th percentiles and median, respectively. Circles and triangles represent individual sera of blood film-negative or blood film-positive individuals, respectively. Whiskers represent minimum and maximum values.

Stratification of the Chonyi cohort revealed age-dependent acquisition of anti-*Pf*spz antibodies (*p* < 0.0001). The age-related increase in prevalence was particularly accentuated among blood film-negative individuals (*p* < 0.001) (Figure 2A). This was in marked contrast with blood film-positive individuals (*p* = 0.72), where prevalence of anti-*Pf*spz responses reached 68% in children younger than 6 years (Figure 2A). In individual age groups, the proportion of *Pf*spz responders was consistently higher among blood film-positive individuals, although this difference was only significant among individuals aged 7–8 years (*p* = 0.001) (Figure 2A).

Despite the presence of several high responders among children, anti-*Pf*spz antibody titers significantly correlated with age (*p* < 0.001) (Figure 2B). This was significant among blood film-negative individuals (*p* = 0.001), but not blood film-positive individuals (*p* = 0.08). Differences in median end titer between the two cohort subsets were non-significant in all age groups.

Acquisition of antibodies against the *Pf*CSP repetitive region (NANP)_5_ occurred in an age-dependent manner (*p* < 0.0001). Anti-(NANP)_5_ antibody acquisition correlated with age among blood film-negative individuals (*p* < 0.001) and blood film-positive individuals (*p* = 0.002) (Figure 2C). The proportion of (NANP)_5_-reactive sera was comparable between blood film-positive individuals and blood film-negative individuals.

There was an age-dependent increase in levels of anti-(NANP)_5_ antibodies, but this increase was not statistically significant (*p* = 0.19) (Figure 2D). Levels of anti-(NANP)_5_ antibodies were comparable between blood film-negative and blood film-positive individuals.

### Correlation of Antibodies against *Pf* Sporozoites with Malaria Incidence

#### Univariate Analysis

In univariate analysis, there was no marked difference in the cumulative risk of clinical malaria depending on *Pf*spz responder status (*p* = 0.19) (Table S2 in Supplementary Material). However, the risk of clinical malaria was significantly reduced among *Pf*spz responders who were blood film positive at the beginning of follow-up (*p* = 0.015). This association was not observed among blood film-negative individuals.

Overall, individuals with a positive antibody response to (NANP)_5_ displayed a significantly lower risk of clinical malaria during follow-up (*p* = 0.006), although this association was only significant among blood film-positive individuals (*p* = 0.03) (Table S2 in Supplementary Material).

#### Survival Analysis

**Figure 3:**
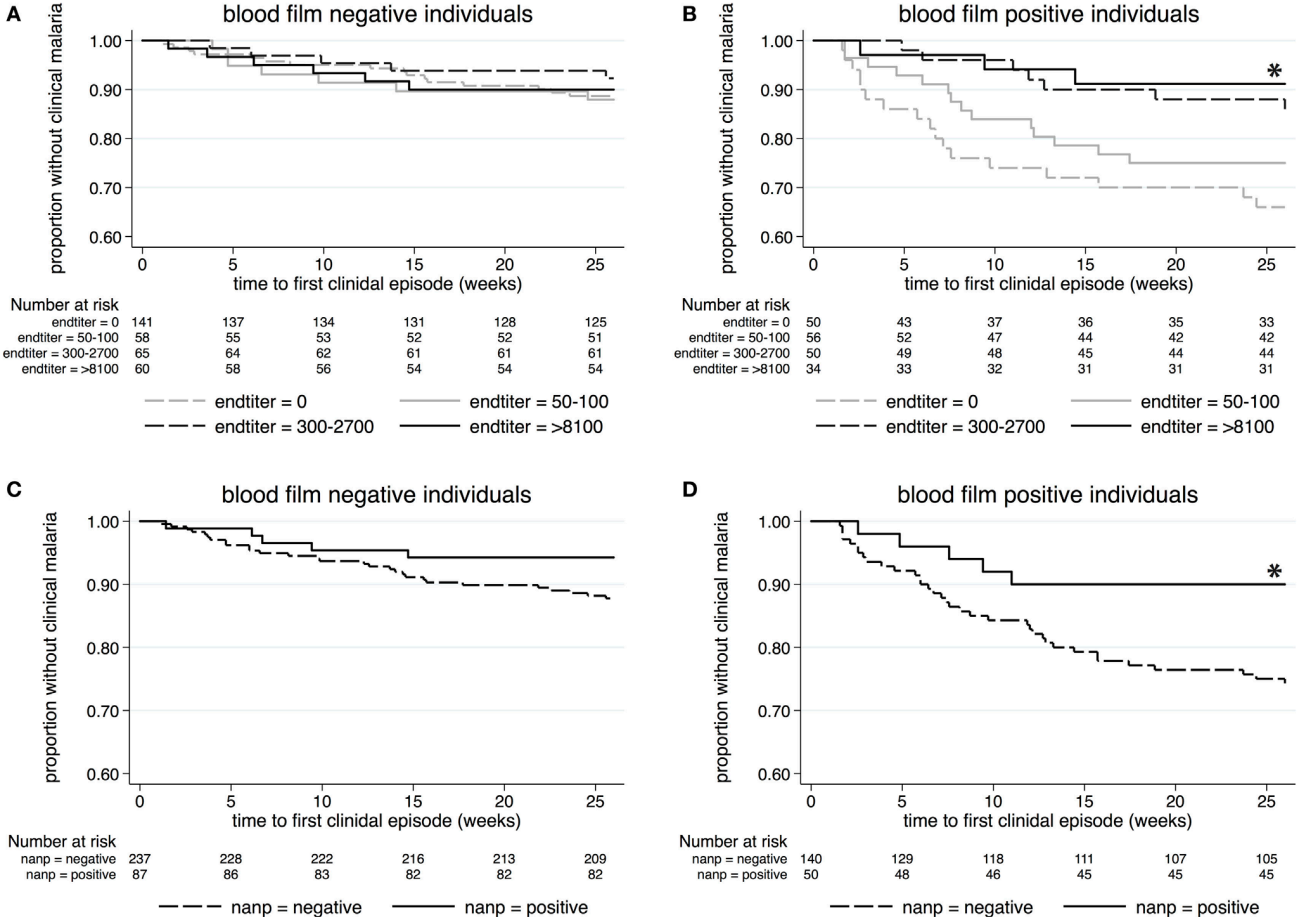
**Kaplan–Meier analysis of time to clinical malaria during the 26-week follow-up**. Survival curves display the proportion of malaria-free individuals over time, stratified by anti-*Pf*spz antibody titer **(A,B)** or (NANP)_5_-seropositivity status **(C,D)**. **(A)** Among blood film-negative individuals, serum levels of anti-*Pf*spz antibodies did not have an impact on the incidence of clinical malaria (log-rank Mantel–Cox test: χ^2^ = 0.81; *p* = 0.847). **(B)** High anti-*Pf*spz antibody titers were significantly associated with a reduced risk of and increased time to first clinical episode among blood film-positive individuals (log-rank Mantel–Cox test: χ^2^ = 10.65; *p* = 0.014). **(C)** Anti-(NANP)_5_ seropositivity did not correlate with malaria incidence among blood film-negative individuals (log-rank Mantel–Cox test: χ^2^ = 2.74; *p* = 0.098). **(D)** Anti-(NANP)_5_ seropositivity was significantly associated with reduced malaria incidence among blood film-positive individuals (log-rank Mantel–Cox test: χ^2^ = 5.02; *p* = 0.025).

According to Kaplan–Meier analysis, antibodies against whole *Pf*spz did not have any impact on clinical malaria incidence among individuals who were blood film negative at the time of serum collection (*p* = 0.85) (Figure 3A). In marked contrast, low (1:50–1:100), moderate (1:300–1:2,700), and high (≥1:8,100) anti-*Pf*spz titers correlated with a reduced risk of and increased time to first (or only) episode of clinical malaria among blood film-positive individuals (*p* = 0.014) (Figure 3B).

(NANP)_5_ responses did not correlate with the incidence of febrile malaria among blood film-negative individuals (*p* = 0.1) (Figure 3C), but (NANP)_5_ seropositivity was significantly associated with the reduced risk of and increased time to clinical malaria among blood film-positive individuals (*p* = 0.025) (Figure 3D).

#### Multivariate Analysis

**Figure 4:**
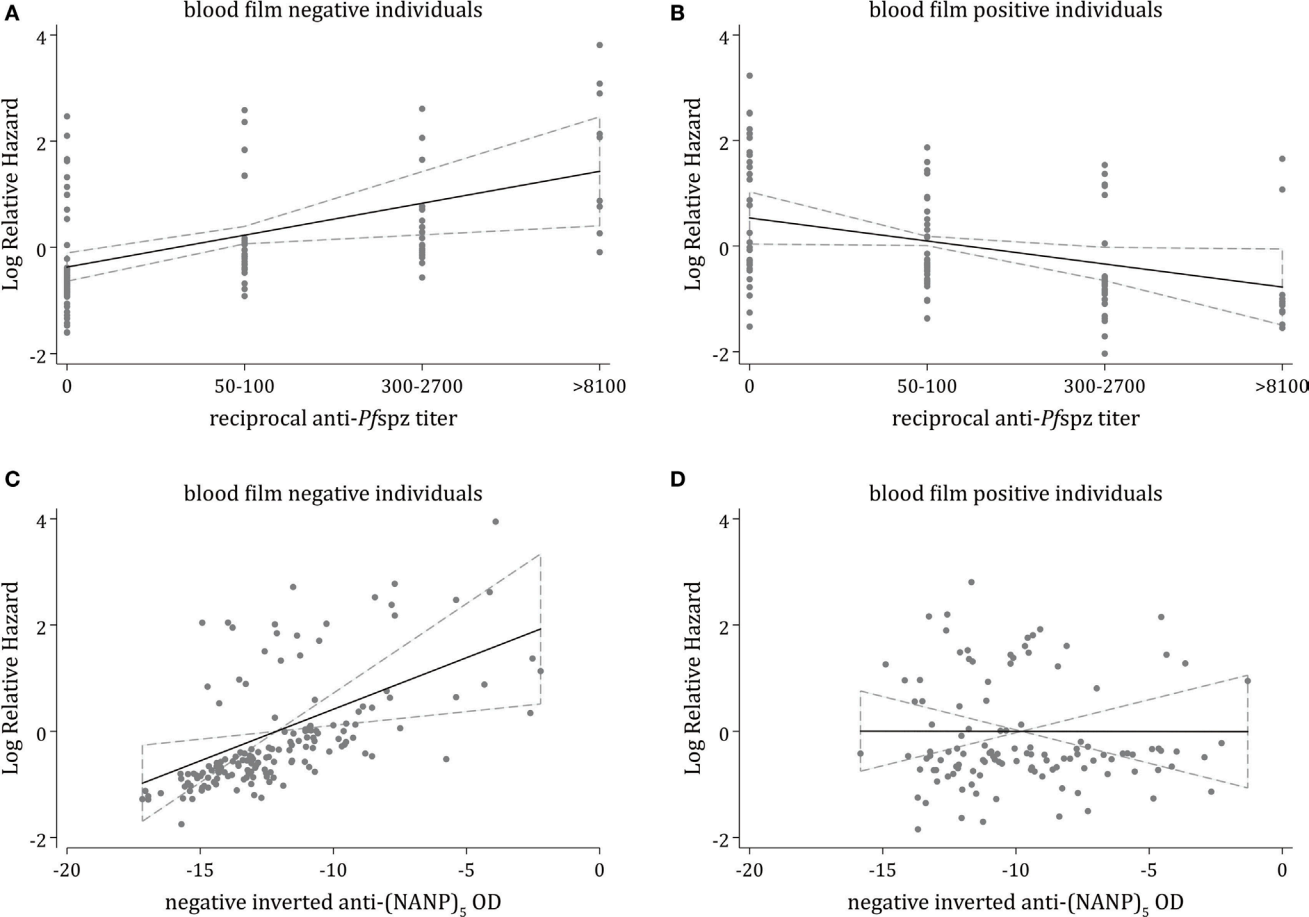
**Multivariable analysis of associations of antisporozoite antibodies against clinical malaria**. Multivariable fractional polynomial Cox regression models estimating the independent effect of different levels of anti-*Pf*spz **(A,B)** or anti-(NANP)_5_ **(C,D)** antibodies on the incidence of clinical malaria during follow-up, stratified by blood film positivity and adjusted for potential confounding variables, including age, malaria exposure, and plasma levels of antibodies against blood stage antigens (MSP1-3, AMA1, EBA175, and *Pf* schizont extract); malaria exposure was measured by the proportion of infected individuals within a 1-km radius of an index case, calculated using weekly slide data for acute episodes, and using the cross-sectional slide data (38). **(A)** Among blood film-negative individuals, increasing anti-*Pf*spz levels were associated with a significantly increased rate of clinical malaria during follow-up [hazard ratio (HR) = 1.82; 95% CI = 1.19–2.81; *p* = 0.006]. **(B)** Among blood film-positive individuals, increasing anti-*Pf*spz levels were associated with a reduced rate of clinical malaria during follow-up, although these results were not statistically significant after applying the Bonferroni correction (HR = 0.65; 95% CI = 0.43–0.97; *p* = 0.035). **(C)** Increasing anti-(NANP)_5_ levels were associated with a significantly increased rate of clinical malaria during follow-up among blood film negatives (HR = 1.21; 95% CI = 1.05–1.40; *p* = 0.007). To reduce the skewness while maintaining the direction of association, anti-(NANP)_5_ optical density (OD) values were transformed to negative inverse values. **(D)** No association between increasing anti-(NANP)_5_ levels and a reduced rate of clinical malaria during follow-up was observed among blood film-positive individuals (HR = 0.99; 95% CI = 0.88–1.13; *p* = 0.992).

To minimize bias through potential confounders, we developed a multivariable polynomial Cox regression model including the three critical covariates (i) age, (ii) malaria exposure, and (iii) plasma levels of antimerozoite stage antibodies. Increasing levels of anti-*Pf*spz antibodies were independently associated with a significantly increased risk of experiencing clinical malaria during follow-up among blood film-negative individuals (*p* = 0.006) (Figure 4A). The model showed a similar trend among anti-(NANP)_5_ responders, with a significantly increased hazard of clinical malaria among blood film-negative individuals (*p* = 0.007) (Figure 4C). Among blood film-positive individuals, increasing levels of anti-*Pf*spz antibodies were associated with a decreased risk of clinical malaria during follow-up, although this association was not significant after the Bonferroni correction was applied (*p* = 0.035) (Figure 4B). Anti-(NANP)_5_ antibody levels were not associated with risk of clinical malaria during follow-up among blood film-positive individuals (*p* = 0.99) (Figure 4D).

#### Interaction Analysis

The relationship between antisporozoite antibodies, microscopically detectable *Pf* parasitemia, and the incidence of clinical malaria was further analyzed through a Cox regression interaction model assessing (i) the effect of antisporozoite antibodies among blood film-negative individuals, (ii) the effect of microscopically detectable *Pf* parasitemia among sporozoite non-responders, and (iii) the effect of interactions between antisporozoite antibody levels and microscopically detectable *Pf* parasitemia on the time to first or only clinical malaria episode during follow-up, independent of age and malaria exposure.

Among blood film-negative individuals, the model indicated an increased hazard of clinical malaria in high *Pf*spz responders, although this association was not significant after the Bonferroni correction (*p* = 0.014) (Table 1). Microscopically detectable *Pf* parasitemia significantly increased the hazard of clinical malaria among individuals who did not react to *Pf*spz (*p* < 0.001). Increasing titers of anti-*Pf*spz antibodies among blood film-positive individuals conferred a strong protection against clinical malaria, independent of age and malaria exposure (*p* = 0.002) (Table 1). Hence, the model indicated a strong interaction between the effect of anti-*Pf*spz antibody levels and *Pf* parasitemia on the risk of clinical malaria. The effect of anti-*Pf*spz antibody levels was protective in blood film-positive individuals and predicted increased risk of first or only clinical episode in blood film-negative individuals. There was a significant difference between the model with and without the interaction term (likelihood ratio test: χ^2^ = 26.05; *p* = 0.0005). A similar model identified an association between increasing anti-(NANP)_5_ antibody levels and microscopically detectable *Pf* parasitemia on the risk of clinical malaria (*p* = 0.001) (Table 2). Again, there was a significant difference between the model with and without interaction term (likelihood ratio test: χ^2^ = 24.38; *p* < 0.0001).

**Table 1:** **Effect of interactions between anti-*Pf*spz antibody levels and microscopically detectable *Pf* parasitemia on the risk of clinical malaria**.

	Hazard ratio (95% CI)	*p* Value
***Pf*spz antibody end titer among blood film-negative individuals**		
0	1	
50–100	1.54 (0.63–3.76)	0.339
300–2,700	1.39 (0.51–3.77)	0.520
8,100–24,300	4.27 (1.34–13.58)	0.014
**Microscopically detectable *Pf* parasitemia in *Pf*spz non-responders**		
bf (−)	1	
bf (+)	5.12 (2.34–11.19)	<0.001*
**Interaction between anti-*Pf*spz antibody levels and microscopically detectable *Pf* parasitemia**		
bf (+)^a^ *Pf*spz (0)	1	
bf (+)^a^ *Pf*spz (50–100)	0.43 (0.14–1.34)	0.146
bf (+)^a^ *Pf*spz (300–2,700)	0.24 (0.06–0.90)	0.035
bf (+)^a^ *Pf*spz (8,100–24,300)	0.07 (0.01–0.37)	0.002*
Age (years)	0.85 (0.76–0.95)	0.005
Exposure index^b^	4.42 (0.89–21.81)	0.068

*Hazard ratios were computed with a logistic Cox regression model with and without one-, two-, or three-way interactions between *Pf*spz antibodies, microscopically detectable blood stage *Pf* infection at the time of serum collection prior to the start of the malaria transmission season, and malaria exposure. The model assesses (i) the effect of *Pf*spz antibody levels among blood film-negative individuals, (ii) the effect of microscopically detectable *Pf* parasitemia among *Pf*spz non-responders, and (iii) the effect of interactions between *Pf*spz antibody levels and microscopically detectable *Pf* parasitemia on the time to first malaria episode during follow-up, independent of age and malaria exposure index*.

*Pfspz, whole Pf sporozoites; Pf, Plasmodium falciparum; bf (−), blood film negatives at the time of serum collection; bf (+), blood film positives at the time of serum collection*.

*Significant at α = 0.05 after Bonferroni correction (critical value α = 0.004).

*^a^Interaction terms*.

*^b^Exposure index: proportion of infected individuals within 1 km of the index case, calculated using weekly slide data for acute episodes only, and using the cross-sectional slide data*.

**Table 2:** **Effect of interactions between anti-(NANP)_5_ antibody levels and microscopically detectable *Pf* parasitemia on the risk of clinical malaria**.

	Hazard ratio (95% CI)	*p* Value
**(NANP)__5__ antibody levels among blood film-negative individuals**		
(NANP)_5_	1.18 (1.05–1.31)	0.004*
**Microscopically detectable ***Pf*** parasitemia per OD increase in anti-(NANP)5-antibody level**		
bf (−)	1	
bf (+)	0.14 (0.03–0.67)	0.014
**Interaction between anti-(NANP)5 antibody levels and microscopically detectable *Pf* parasitemia**		
bf (+)^a^ (NANP)_5_	0.77 (0.66–0.89)	0.001*
Age (years)	0.83 (0.74–0.95)	0.004*
Exposure index^b^	3.57 (0.68–18.8)	0.132

*Hazard ratios were computed with a logistic Cox regression model with and without one-, two-, or three-way interactions between anti-(NANP)_5_ antibodies, microscopically detectable blood stage *Pf* infection at the time of serum collection prior to the start of the malaria transmission season, and malaria exposure. The model assesses (i) the effect of anti-(NANP)_5_ antibody levels among blood film-negative individuals, (ii) the effect of microscopically detectable *Pf* parasitemia per OD increase in anti-(NANP)_5_ antibody level, and (iii) the effect of interactions between anti-(NANP)_5_ antibody levels and microscopically detectable *Pf* parasitemia on the time to first malaria episode during follow-up, independent of age and malaria exposure index*.

*(NANP)_5_, immunodominant icosapeptide repeats of *Pf* circumsporozoite protein; OD, optical density; bf (−), blood film negatives at the time of serum collection; bf (+), blood film positives at the time of serum collection; Pf, Plasmodium falciparum*.

*Significant at α = 0.05 after Bonferroni correction (critical value α = 0.004).

*^a^Interaction terms*.

*^b^Exposure index: proportion of infected individuals within 1 km of the index case, calculated using weekly slide data for acute episodes only, and using the cross-sectional slide data*.

## Discussion

In this study, we analyzed the association of naturally acquired antibody responses against whole *Pf*spz and the *Pf*CSP CRR (NANP)_5_ with microscopically detectable *Pf* parasitemia and clinical malaria in the longitudinally monitored Chonyi cohort. The data indicate that robust antisporozoite immune responses can be naturally acquired early in life and reveal a potentially protective interaction between the effect of anti-*Pf*spz antibodies and microscopically detectable *Pf* parasitemia on the risk of uncomplicated malaria.

### Naturally Acquired Humoral Immunity against *Pf* Sporozoites

In good agreement with previous studies (15, 39), prevalence and intensity of antibody responses to *Pf*spz and (NANP)_5_ were correlated within the Chonyi cohort. However, even among high *Pf*spz responders, some sera remained (NANP)_5_ negative. Slow acquisition of anti-(NANP)_5_ antibodies has already been reported (17). Previous studies demonstrated that endemic sera retain IFA signals despite depletion of anti-(NANP)*_n_* antibodies by the cognate peptide (39). Thus, the sera used in this study may have reacted to CSP antigens other than (NANP)_5_ or non-CSP epitopes present internally or on the sporozoite surface (40), suggesting that exclusive quantification of anti-(NANP)*_n_* titers may largely underestimate the prevalence and breadth of naturally acquired preerythrocytic humoral immune responses. This discrepancy may be, at least partly, explained by different sensitivities of the assays used. In particular, the use of air-dried, acetone fixed sporozoites may inactivate some epitopes on the sporozoite  surface and instead expose others that are usually inaccessible to protective antibodies. However, since our serology data show that fixed sporozoites are well recognized in IFA, we anticipate that live sporozoites tested in follow-up studies will likely display comparable activity.

In this study, we confirmed an age-dependent pattern of anti-*Pf*spz antibody acquisition (9–11), although we also identified several high responders among children, whose sera reacted to *Pf*spz at dilutions of up to 1:24,000. This is remarkable, considering that virtually all sera from adult volunteers immunized five times intravenously with ~135,000 metabolically active, irradiation-attenuated *Pf* sporozoites recognized *Pf*spz at ≤1:7,000 in a similar IFA assay (41). This would indicate that naturally acquired immune recognition of *Pf*spz fundamentally differs from syringe-injected, purified, and cryopreserved parasites.

Especially among younger age groups, the prevalence of antisporozoite responders was consistently higher among blood film-positive subjects. Whether the increased reactivity against sporozoites in this group could be attributable to recent or recurrent exposure to *Pf* could not be inferred based on the available data. Since the presence of asymptomatic *Pf* infection was determined by microscopy, we cannot exclude that a significant proportion of blood film-negative individuals may have carried blood stage parasites below the microscopy detection level. In future studies, more sensitive methodologies, such as quantitative polymerase chain reaction or loop-mediated isothermal amplification, may provide a more accurate estimate of the prevalence of asymptomatic *Pf* infection among study participants.

Despite the overall lower reactivity, an age distribution of the prevalence of anti-(NANP)_5_ antibodies was detectable in this cohort, confirming previous findings (14, 15, 42). Microscopically detectable *Pf* parasitemia did not seem to affect acquisition of the anti-(NANP)_5_ antibody repertoire, corroborating previous observations that anti-(NANP)_5_ antibodies do not necessarily correlate with exposure (43, 44). On the other hand, several studies showed that anti-(NANP)*_n_* antibody responses fluctuate with seasonal or geographical transmission variation (13, 16), and outbreak investigations showed that a single sporozoite inoculation is able to induce humoral immunity against (NANP)*_n_* (15, 18). These conflicting reports may reflect epidemiological differences between the geographically distinct study areas.

Together, these observations support the notion of naturally induced acquisition of antibodies against the whole sporozoite and, to a lesser extent, the dominant repeat region of its main surface antigen. Whether the prevalence and intensity of anti-*Pf*spz responses may represent a valuable marker for recent and/or repeated exposure remains to be elucidated in further longitudinal cohort studies, which should include entomological data to estimate the temporospatial dynamics of sporozoite transmission. Additional immune markers are needed to ultimately disentangle recent and past *Plasmodium* exposures.

### Potentially Protective Associations of Antisporozoite Antibodies with Clinical Malaria

Previous investigations in the Chonyi cohort have shown that the correlation of naturally acquired antibodies to blood stage antigens with the risk of disease varies depending on the presence of microscopically detectable *Pf* infection (31, 34, 45, 46). Therefore, the relationship of antisporozoite antibodies with malaria incidence was analyzed separately in individuals who were blood film positive or blood film negative at sampling.

Our analysis revealed dual features of antisporozoite antibodies. Among individuals who were parasite free or carrying *Pf* parasites below the level of detection by microscopy at the start of the malaria transmission season, high anti-*Pf*spz and anti-(NANP)_5_ antibody titers were significantly correlated with increased hazard of clinical malaria during follow-up. Acute antibody responses against the sporozoite are most likely elicited upon *Pf* inoculation. Blood film-negative individuals displaying higher antisporozoite antibody titers may have been recently exposed to a strong inoculation dose and, as a consequence, may be at higher risk of clinical malaria. Since our study only assessed humoral immune responses against the NF54 *Pf* strain, we cannot formally exclude that these individuals may have been infected by novel *Pf* strains to which they were immunologically unresponsive.

In marked contrast, higher levels of anti-*Pf*spz antibodies predicted a lower risk of and delayed progression to clinical malaria among those individuals who were blood film positive at the start of the malaria transmission season. Notably, we detected a strong and potentially protective interaction between the effect of both anti-*Pf*spz and anti-(NANP)_5_ antibody levels and microscopically detectable *Pf* parasitemia at the start of the transmission season on the risk of clinical malaria, which was independent of age and *Pf* exposure. The interaction analysis showed that the potentially protective effect of antisporozoite antibodies was greater among individuals with detectable baseline *Pf* parasitemia than those who were blood film negative at the beginning of the malaria transmission season. Because of this interaction, blood film-negative and -positive individuals displaying high antibody titers against sporozoites may experience different risks of clinical malaria. In contrast, the difference in risk of disease may be comparatively similar among blood film-negative or -positive individuals who display low antisporozoite titers. At higher antisporozoite antibody levels, blood film-positive individuals had significantly lower risk of malaria compared with blood film-negative individuals. This correlation was previously unrecognized and provides a rationale for future studies on the role(s) of preerythrocytic immunity in partial protection against malaria. Although highly speculative, one possibility is that members of the Chonyi cohort may have been infected by a diverse range of *Pf* strains, which in turn could have different immunological and pathogenic properties, thus influencing both individual immune responses and the patterns of clinical malaria incidence during the follow-up period. In such a scenario, selection through protective effects of antisporozoite antibodies could favor alternative strains.

One major limitation of this analysis is that data on participants’ *Pf* carrier status were only available from a cross-sectional measurement at the start of the malaria transmission season. It is plausible that the status of single individuals may have changed during the follow-up. Such changes may have affected the patterns of malaria incidence in the cohort over time, but this could not be taken into account in this analysis.

Although naturally induced antisporozoite immunity does not translate into sterile protection, partial preerythrocytic immunity might play a significant role in protection from clinical symptoms (28, 29). However, frequent inoculations over a longer time period might be required to induce short-lived, potentially protective anti-*Pf*spz antibodies. While such requirements might not have been met in the blood film-negative subset, the identification of children with high *Pf*spz antibody titers among the blood film-positive individuals indicates that high and potentially protective threshold levels may be acquired early in life. These hypotheses are based on the findings from this data set, but do not take into account the variability of the data. It is possible that other serologic data sets based on different cohorts or transmission settings may deliver contrasting results.

We cannot formally exclude that additional factors may have confounded this analysis. For instance, multiclonality of asymptomatic *Pf* infections correlates with a reduced risk of subsequent clinical malaria (47–49), and this association is dependent on the intensity of transmission (50). In children, sickle cell trait is independently associated with a delayed time to first clinical episode (51). Since these factors were not accounted for in our analysis, residual confounding may have occurred.

In addition, it is possible that the anti-*Pf*spz antibodies detected by IFA may actually recognize antigens that are shared between sporozoites and erythrocytic stages (52). Potentially protective anti-*Pf*spz antibodies detected in this study may be (i) antibodies that have been generated by blood stage infection but are also effective against sporozoites or (ii) antibodies that are effective against blood stages, in which case the IFA assay may have served as a proxy of protective blood stage responses.

Although there is currently scarce evidence that reducing the sporozoite load may reduce clinical malaria, possible scenarios for the contribution of antisporozoite antibodies to protection from clinical malaria include (i) reduction of sporozoite numbers by opsonization and subsequent phagocytosis through innate immune cells and (ii) blocking of essential parasite ligands for liver invasion receptors, as suggested by reduced sporozoite invasion of human liver cell cultures *in vitro* following the addition of endemic sera (53). Both effects may ultimately limit the scale of liver infection and, consequently, delay or mitigate the emergence of liver merozoites that initiate blood stage infection. Although the NANP repeats of CSP represent an immunodominant B-cell epitope (37), it is tempting to speculate that only broad recognition of multiple sporozoite antigens may represent a good marker for recent exposure and elicit protection against subsequent malaria incidence.

## Conclusion

Observations in Kenya and other endemic areas indicated that only a very small fraction of mosquito inoculations results in reappearance of malaria after drug treatment (54–56). While these observations might be partly attributed to potent blood stage immune responses or inefficient sporozoite inoculation by infected mosquitoes, they could also suggest that malaria-exposed individuals are able to develop partially protective immunity against sporozoites. In this study, whole *Pf* sporozoites were employed for the first time as target antigen in a longitudinal study to assess the role(s) of naturally acquired antibodies in protective immunity against clinical malaria. A potentially protective contribution of antisporozoite antibodies was detected, suggesting that preerythrocytic immunity might indeed contribute to naturally acquired protection and that antibodies against several sporozoite antigens might be superior than responses to single antigens. Several obstacles complicate the interpretation of naturally acquired antibodies against sporozoites and their potentially protective roles. First, assessment of the relationship between disease and immune responses that never reach sterile protection is complex. In addition, endpoints in *Pf* malaria include microscopic detection of blood stage infection, while preerythrocytic stages remain undetectable. Additional functional assays, such as sporozoite invasion inhibition and antibody-dependent, cell-mediated immunity, will add further confidence to whether high antisporozoite titers can effectively reduce the burden of this vector-borne parasitic disease and exert a hitherto neglected benefit in preventing clinical malaria episodes.

## Ethics Statement

Ethical approval was obtained from the Kenya National Research Ethics Committee (study protocol number REF CTMDR/SCC/1340). All participants (or parents/guardians of children aged ≤14 years) gave written informed consent (31, 34, 36). The local dialect was used to explain the study protocol to the participants, and a copy of the consent form was left in each household, so that all family members could review it prior to consenting.

## Author Contributions

All authors delivered substantial contributions to the conception or design of the work or the acquisition, analysis, or interpretation of data for the work; contributed to drafting the work or revising it critically for important intellectual content; gave final approval of the version to be published; agreed to be accountable for all aspects of the work in ensuring that questions related to the accuracy or integrity of any part of the work are appropriately investigated and resolved.

## Conflict of Interest Statement

This research was conducted in the absence of any commercial or financial relationships that could be construed as a potential conflict of interest. The authors did not at any time receive payment or services from a third party for any aspect of the submitted work. There are no financial relationships with entities that could be perceived to influence or that give the appearance of potentially influencing, what is written in the submitted work. There are no patents and copyrights, whether pending, issued, licensed, and/or receiving royalties that are relevant to this work. All the authors declare no conflict of interest.

## Abbreviations

*Pf, Plasmodium falciparum*; *Pf*spz, whole *Plasmodium falciparum* sporozoites; CSP, circumsporozoite protein (major sporozoite surface protein); (NANP)_5_, immunodominant icosapeptide repeats of *Pf* circumsporozoite protein; CRR, central repeat region of circumsporozoite protein; MSP1-3, merozoite surface proteins 1-3; AMA1, apical membrane antigen-1; EBA-175, erythrocyte binding antigen 175.
