# Supplementary material for: High Sporozoite Antibody Titers in Conjunction with Microscopically Detectable Blood Infection Display Signatures of Protection from Clinical Malaria
Source: Front Immunol. 2017 May 8;8:488. doi: 10.3389/fimmu.2017.00488 (PMC5421148; doi:10.3389/fimmu.2017.00488)
Supplement: Supplementary file 1 [file data_sheet_1.docx]

Supplementary material for:

**High Sporozoite Antibody Titers in conjunction with Microscopically Detectable Blood Infection Display Signatures of Protection from Clinical Malaria**

Vittoria Offeddu, Ally Olotu, Faith Osier, Kevin Marsh, Kai Matuschewski, and Vandana Thathy

**Content:**

Supplementary tables S1 and S2

**Table S1. Cumulative incidence of clinical malaria in the Chonyi cohort according to age category and blood film positivity.** Proportion of individuals developing at least one episode of clinical malaria during follow-up, stratified by age group and microscopically detectable *Pf* infection at the beginning of the *Pf* transmission season, as assessed by thin and thick blood films. Overall, the cumulative incidence of clinical malaria decreased with age (Pearson’s χ2: 67.6; p<0.0001) and was higher among blood film positive individuals compared to blood film negatives (Pearson’s χ2: 11.8; p=0.001). In individual age groups, this difference was significant among children aged 1-4 (Pearson’s χ2: 3.8-21.2; p≤0.05) and 9-10 years (Pearson’s χ2: 3.9; p=0.049). Clinical infections were shifted to slightly older age groups among blood film negative children.

**≥1 episode of clinical malaria during follow-up***

**Age group bf (+) whole cohort bf (+) bf (-)**

**[Years] n (%) n (%) n (%) n (%) n (%)**

1/2 52 (10%) 9 (17%) 14 (27%) 8 (89%) 6 (14%)

3/4 56 (11%) 23 (41%) 23 (41%) 13 (57%) 10 (30%)

5/6 51 (10%) 25 (49%) 12 (24%) 4 (16%) 8 (31%)

7/8 60 (12%) 22 (37%) 11 (18%) 5 (23%) 6 (16%)

9/10 60 (12%) 38 (63%) 6 (10%) 6 (16%) 0 (0%)

11 – 15 95 (19%) 47 (49%) 8 (8%) 5 (11%) 3 (6%)

16 – 30 58 (11%) 14 (24%) 0 (0%) 0 (0%) 0 (0%)

31 – 50 54 (10%) 9 (17%) 0 (0%) 0 (0%) 0 (0%)

> 50 28 (5%) 3 (11%) 1 (4%) 0 (0%) 1 (4%)

all 514 (100%) 190 (37%) 75 (15%) 41 (22%) 34 (11%)

* incidence of uncomplicated clinical malaria during the 26-week follow-up among i) the whole cohort, ii) individuals who were blood film positive at the start of the malaria transmission season or iii) individuals who were blood film negative at the start of the malaria transmission season

bf (+) = blood film positive individuals, as determined during the cross-sectional bleed at the start of the malaria transmission season

bf (-) = blood film negative individuals, as determined during the cross-sectional bleed at the start of the malaria transmission season

**Table S2. Univariate analysis of associations of anti-sporozoite antibodies with clinical malaria**

Crude risk ratios for developing at least one episode of clinical malaria during the 26-week follow-up among subjects who mounted a humoral immune response to *Pf* sporozoites, compared to individuals whose sera did not contain anti-sporozoite antibodies at microscopically detectable levels at the start of follow-up. Responses to whole *Pf* sporozoites (*Pf*spz) or the central repeat region of the circumsporozoite protein were measured by immunofluorescence assay or (NANP)_5_-ELISA, respectively. The analysis was conducted with the whole cohort (n=514) or by *Pf* blood film positivity.

**cohort** **mal (+)^a^ mal (-)^b^ Risk Ratio p-value**

*Pf*spz - 33 158 1

*Pf*spz + (all) 42 281 0.72 (0.44-1.18) 0.186

*Pf*spz + (50-100) 21 93 1.08 (0.59-1.98) 0.800

*Pf*spz + (300-2700) 12 103 0.56 (0.28-1.13) 0.105

*Pf*spz + (≥1:8100) 9 85 0.51 (0.23-1.11) 0.089

----------------------------------------------------------------------------------------------------------------------------

(NANP)_5_ - 65 312 1

(NANP)_5_ + 10 127 0.38 (0.19-0.76) **0.006***

**blood film negative individuals** **mal (+)^a^ mal (-)^b^ Risk Ratio p-value**

*Pf*spz- 16 125 1

*Pf*spz + (all) 18 165 0.85 (0.42-1.74) 0.661

*Pf*spz + (50-100) 7 51 1.07 (0.42-2.77) 0.885

*Pf*spz + (300-2700) 5 60 0.65 (0.23-1.86) 0.424

*Pf*spz + (≥1:8100) 6 54 0.87 (0.32-2.34) 0.780

----------------------------------------------------------------------------------------------------------------------------

(NANP)_5_ – 29 208 1

(NANP)_5_ + 5 82 0.44 (0.16-1.17) 0.100

**blood film positive individuals** **mal (+)^a^ mal (-)^b^ Risk Ratio p-value**

*Pf*spz- 17 33 1

*Pf*spz + (all) 24 116 0.40 (0.19-0.84) **0.015***

*Pf*spz + (50-100) 14 42 0.65 (0.28-1.50) 0.312

*Pf*spz + (300-2700) 7 43 0.32 (0.12-0.85) **0.023***

*Pf*spz + (≥1:8100) 3 31 0.19 (0.05-0.71) **0.013***

----------------------------------------------------------------------------------------------------------------------------

(NANP)_5_ – 36 104 1

(NANP)_5_ + 5 45 0.32 (0.12-0.87) **0.026***

^a^ Number of individuals experiencing at least one episode of clinical malaria during follow-up

^b^ Number of individuals who remained malaria-free during follow-up

*Pf*spz + / *Pf*spz -: individuals who did or did not respond to whole air-dried *P.falciparum* sporozoites (*Pf*spz) by immunofluorescence assay (IFA); anti-*Pf*spz antibody levels were stratified by reciprocal anti-sporozoite end titer, defined as the last dilution at which each serum responded to *Pf*spz by IFA;

(NANP)_5_ + / (NANP)_5_ -: individuals who did or did not recognize the (NANP)_5_-peptide by enzyme-linked immunosorbent assay (ELISA); the seropositivity cut-off was defined as mean OD + 3 standard deviations from 20 pooled sera from unexposed individuals.

* Significant at α=0.05
